# Safeguarding Against Abuse in Religious Contexts: Challenges and Potential in the Relationship Between Statutory Services and Faith-Based Organizations

**DOI:** 10.1007/s11089-025-01299-z

**Published:** 2026-02-16

**Authors:** Moira E. Lafferty, Justin Humphreys, Katy Jackson, Leigh McFarlane, Timothy Cartwright

**Affiliations:** 1 University of Chester, Parkgate Road, Chester, CH1 4BJ UK; 2 Thirtyone:eight, College Road, Hextable Swanley, UK

**Keywords:** Statutory services, Faith-based organizations, Safeguarding, Relationships

## Abstract

Faith-based organizations (FBOs) are integral to community social structures, providing services that often extend beyond spiritual guidance. Due to their longstanding presence and trust within communities, FBOs are uniquely positioned to contribute to safeguarding efforts against abuse. Despite their potential, research indicates that there is a degree of ineffective collaboration between FBOs and statutory services (SSs), particularly in the context of safeguarding vulnerable individuals. The established literature suggests that many FBO safeguarding leads tend to be unaware of broader safeguarding policies and how to discuss concerns with SSs. The present study therefore explores the relationship between FBOs and SSs, aiming to identify factors that facilitate or hinder effective collaboration. Through an online open-ended questionnaire, data were gathered from 89 participants, including safeguarding leads in FBOs and professionals from SSs. Thematic analysis revealed four key themes: understanding and supporting spirituality, safeguarding awareness and challenges, communication and collaboration barriers, and suggestions for improved partnership. Helpfulness and understanding of spirituality by SSs led to positive experiences. Conversely, negative experiences stemmed from a lack of understanding of FBOs’ safeguarding roles, poor information sharing, and complex SSs processes. Participants suggested joint training and increased open communication to improve collaboration. The findings emphasize the need for context-specific training and respectful interorganizational (or interagency) working to foster better relationships between FBOs and SSs. Enhancing these partnerships is crucial for effective safeguarding and protection of vulnerable individuals within faith communities. Future research could delve into these suggestions in more depth and further explore the perspectives of SSs to provide a more comprehensive understanding of these relationships. A more comprehensive understanding of the relationship between statutory bodies and those in voluntary safeguarding positions in faith settings.

## Introduction

Faith-based organizations (FBOs) play an influential role in societies worldwide, providing vital support at a local and sometimes international level. Defined by Clarke and Jennings (2008) as “any organization that derives inspiration and guidance for its activities from the teaching and principles of the faith or from a particular interpretation or school of thought within the faith” (p. 6), FBOs include places of worship, faith-based charities, schools, and community networks, to name but a few (Fiddian-Qasmiyeh, 2015). These organizations often provide vital social, educational, and humanitarian services, particularly in and for communities where provision may be limited (Tomalin, 2012), and they often work with children, young people, and vulnerable adults and should therefore have in place measures to prevent harm and respond to safeguarding concerns. While the practicalities and implementation of safeguarding measures differ country to country, in the United Kingdom FBOs usually have a designated safeguard lead whose role includes consulting with SSs (Trend, 2025).

United Kingdom SSs are public services that local authorities and government bodies legally must provide to prevent harm, protect those at risk, and work in partnership with families and voluntary organizations to keep people safe. These include child protection and safeguarding services delivered by Children’s Services, adult social care, the National Health Service (NHS), the police, and education authorities (HM Government, 2023), who should in the case of safeguarding issues work together through multiagency collaboration (Ball et al., 2024).

Effective safeguarding relies on multiagency collaboration to challenge abuse and achieve positive outcomes (Carter et al., 2007; HM Government, 2023; Laming, 2003; Munro, 2011; Romeo, 2015). Morrison (2000) discussed how the U.K. government formulated policy guidelines establishing best practice frameworks for collaborative efforts with the goal of safeguarding vulnerable individuals within the community, for example, Every Child Matters, Working Together to Safeguard Children guidance and The Care Act statutory guidance to increase information sharing and joint decision making between agencies (Department for Education & Skills, 2003; HM Government, 2014, 2023). While the legislation is clear, the implementation of multiagency working, where differing organisations work together collaboratively to meet an individual's or a group's needs, has been more complex. This is partly due to the adoption of differing models of governance and structures in different areas (Ball et al., 2024; McManus & Boulton, 2020). Within England, the most prominent model is the Multi-Agency Safeguarding Hub (MASH; Home Office, 2014). MASH hubs bring together key statutory safeguarding partners to ensure the sharing of information between agencies occurs in a timely manner, assessment of concerns, and putting in place of interventions through a joined-up response (Crockett et al., 2013).

Shorrock et al. (2020) conducted interviews with practitioners from one MASH hub in the north of England; while they found evidence of good practice and improved information sharing, there were still areas for development. For example, while services were all in one space, internal hierarchical structures within each service led to feelings of power imbalance between services. Furthermore, while information sharing had improved, a challenge remained as to the usefulness of the information shared and factors such as service protocols, sharing agreements, and consent, which were cited as barriers. These are not new findings. They echo those found by Atkinson et al. (2005), who examined multiagency working in U.K. local education authorities. Critically, Atkinson et al. also raised communication as a challenge and identified how working at differing levels impacted, for example, those in strategic roles versus those in operational levels. Multiagency collaboration can become even more difficult when the interagency relationships transcend the statutory and voluntary sector divide, especially when considering specific organizations such as FBOs.

Although the Working Together to Safeguard Children (HM Government, 2023) guidance emphasizes the need for all organizations to work in partnership with statutory agencies to protect children from harm, it has only two paragraphs addressing safeguarding within a faith context. Indeed, the attention given to faith settings has reduced incrementally in each revision since 2010. This means that limited guidance and literature on the interaction between FBOs and statutory services (SSs) for multiagency working exists. O’Neill et al. (2010) conclude that there is “a disconnect between social services and faith communities concerning child abuse prevention efforts” (p. 381). More recently, the Independent Inquiry into Child Sexual Abuse (IICSA) has underscored the need for improved collaboration and understanding between SSs and FBOs to effectively address safeguarding concerns (IICSA, 2020). The relationship between FBOs and SSs is pivotal in ensuring the safety and well-being of vulnerable individuals within communities, including children, vulnerable adults, and partners (Morrison, 2000).

FBOs often play a crucial role in the social structure of communities, offering a range of services that extend beyond spiritual guidance to include increased social capital for members (Hepworth & Stitt, 2007), health and well-being initiatives (Meads & Lees, 2018), and support for those in need (Furness & Gilligan, 2012). FBOs often have a long history and ongoing presence within the community and therefore are trusted and viewed as places of safety and security (Bielefeld & Cleveland, 2013). This is especially pertinent in the context of the United Kingdom’s diverse and multicultural society, where FBOs engage with groups that may include individuals from multicultural or marginalized areas of society who may be underserved or overlooked by SSs (Crisp, 2014). In the United Kingdom, the role of safeguarding leads within FBOs has gained increasing attention due to the unique position these organizations hold in providing support and protection to their members. However, for those individuals to be truly supported, there needs to be interagency cooperation between the FBO’s safeguarding lead and external bodies.

Although undertaken in a U.S. context, a study by O’Neill et al. (2010) into the prevention of child abuse and neglect through church and social service collaboration considered the relationship between FBOs and SSs as a useful point of reference. This study highlighted the desire of both FBOs and SSs to work together but, due to the longstanding lack of collaboration, concluded that there is a separation between the desire and the fruition of such work. O’Neill et al. (2010) cite the following factors as reasons for this: lack of time, trust, understanding, and open dialogue and reluctance and a misunderstanding of the role of the church. The study concludes that an effective child abuse prevention strategy should be comprised of FBO and SS leaders working together with shared values, clear goals and responsibilities, and accountability in the partnerships (O’Neill et al., 2010).

In the United Kingdom more recently, Oakley et al. (2016) undertook a review into the understanding of safeguarding adults and vulnerabilities within Christian FBOs. They also emphasized that definitions and recommendations did not work within the faith-based context due to the complexity of faith-based relationships and that, because of this, further work is needed in this area to consider definitions of vulnerability and risk to offer broader understanding and support to adults in faith-based contexts. Moreover, Pentaris (2023), in a qualitative study exploring how U.K. social workers integrated service users’ beliefs and social identities into interactions, found that there was a strong premise that an individual’s beliefs, spiritual view, and religious perspective were important at critical points in service use (initial assessments, conditional intervention, referrals, and child protection issues). However, the study also referenced “religious illiteracy” (p. 40) and the adoption of what was termed avoidant or utilitarian practice approaches (Pentaris, 2023). Gilligan’s (2009) review of religious beliefs and child protection safeguarding further highlights the impact of faith illiteracy and emphasizes that daily practice remains dependent on individual SS staff perceptions of and/or lack of training around FBOs.

In addition, Oakley et al. (2019) completed a study of child abuse linked to faith and belief to understand the experiences of frontline practitioners’ awareness of this type of child abuse. Through this work, they highlighted the importance of understanding faith and belief and limiting negative perceptions of FBOs to increase frontline FBO practitioners’ confidence in and trust of SSs. Moreover, the models of good practice referenced within this study were underpinned by dialogue between SSs and FBOs that diminished distrust and allowed for the building of good relationships (Oakley et al., 2019). A study by Sidebotham et al. (2016) found that safeguarding faith-based leads frequently encounter barriers such as misunderstandings of their roles, lack of communication, and differing cultural contexts.

These challenges are not confined to child safeguarding. Research by Oakley et al. (2016) highlights similar issues in protecting vulnerable adults and spouses within faith communities. Their work indicates that FBOs often lack the necessary support and understanding from SSs, which can lead to significant gaps in safeguarding vulnerable adults and addressing abuse. This suggests that there is a need to explore the processes in place that facilitate collaborative working. One method of doing this would be through the lens of normalization process theory (NPT) (C. May & Finch, 2009). NPT is a framework that explains how new practices become routinely established and embedded within their social and organizational contexts. Originally developed within health care (C. May, 2006), it has subsequently been used to explain social behavior in a variety of settings and to examine working relationships. Central to NPT are four mechanisms: coherence, cognitive participation, collective action, and reflexive monitoring. Through coherence, those within the system make sense of the working practice. Cognitive participation focuses on both engagement and commitment from all parties, collective action refers to operational collaboration where identified barriers hinder, and collective collaboration enhances partnership work. Finally, reflexive monitoring involves assessing how well things are working, and for partnerships, this means ongoing reflection, feedback, and adaptation (C. May & Finch, 2009).

Although some literature highlights the importance of multiagency working between SSs and FBOs, exploration of what works well and where improvements can be made to enhance the relationship is limited. Using an exploratory descriptive design (Hunter et al., 2019), this study aims to firstly explore factors that facilitate or hinder effective collaboration between SSs and FBOs and secondly identify the critical aspects required to enhance the multiagency working relationship in practice.

## Method

### Design and questionnaire development

**Fig. 1:**
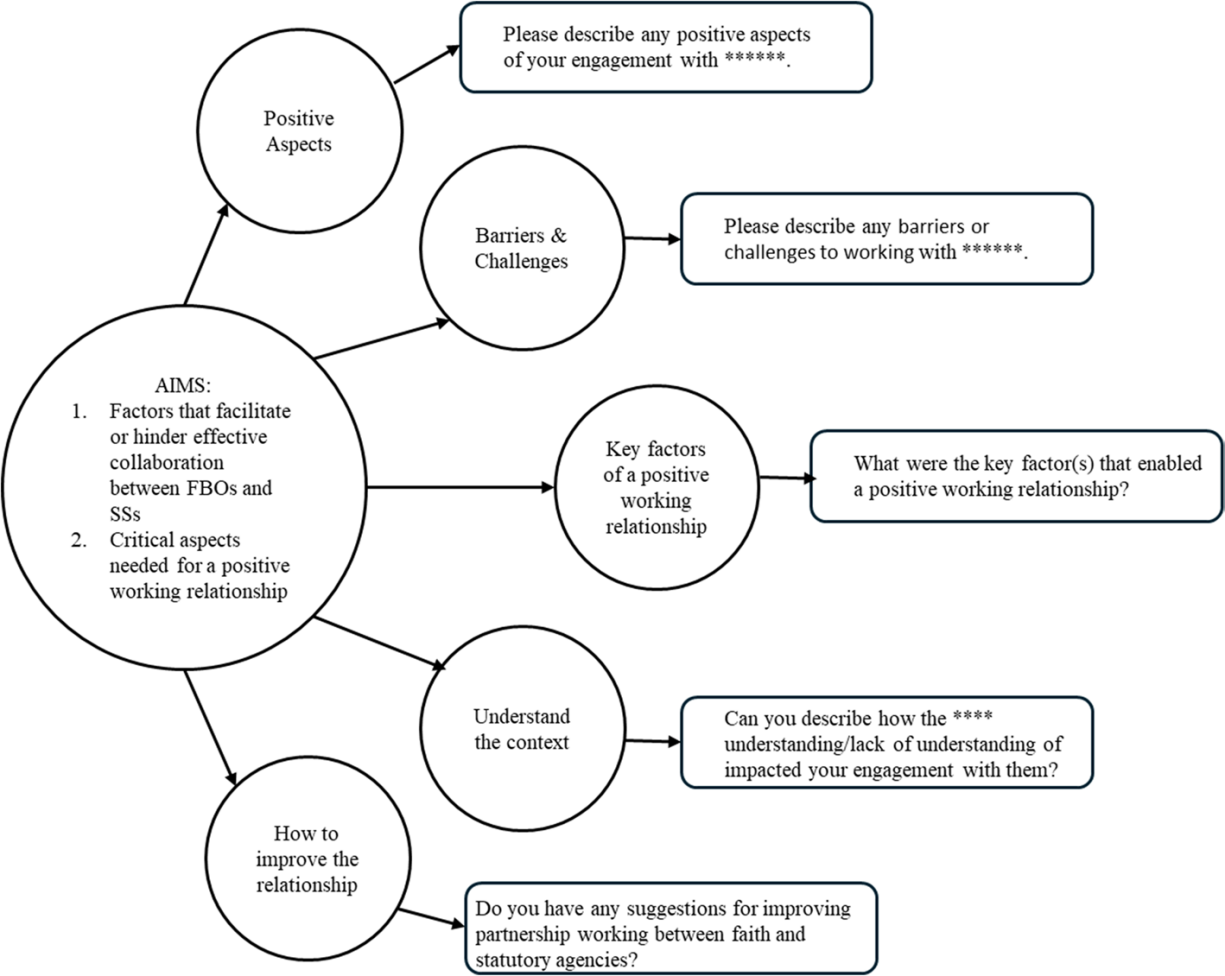
Themes forming the question basis and the questions. Note: *** represents space to allow context specificity. FBO = faith-based organization, SS = statutory service

This qualitative study adopted an online open-ended questionnaire approach to gather data on how those in statutory agencies and faith-based safeguarding leads viewed their working relationship. To develop the questionnaire, we used the aim of the study as an umbrella term, and the research team compiled ideas following the concept of the rational method of survey design (Oosterveld et al., 2019). Given that the research team included academic researchers with strong theoretical knowledge and experts in the field of safeguarding in faith contexts, a broad range of areas emerged (Fig. 1).

These themes allowed for the development of five open-ended questions that encapsulated both perception (e.g., Do you have any suggestions for improving partnership working between faith and statutory agencies?) and experience-based views (e.g., What were the key factors that enabled a positive working relationship?) (Gray, 2009). We added a sixth question on overall experience to capture any general experiences not covered in the five themed questions. To ensure inclusiveness, we used broad wording so that participants from any faith background or none could answer (Fig. 1). We then wrote two versions of each question so that they were contextually specific for the participant group. For example, What was your overall experience of how faith communities engaged with you and the service you work for? (SSs) changed to What was your overall experience of how statutory services engaged with you and your organization? (FBOs). Adapting questions in this manner increases engagement and subsequently the depth and detail of the response (Dillman et al., 2014). The lead author’s School Ethics Board granted ethical approval.

### Recruitment strategy

A purposive convenience sampling strategy was utilized; contact lists for the United Kingdom already established by the research team were used (convenience), and participants needed to meet the inclusion criteria of holding a safeguarding role within an FBO or working in statutory service (purposive) (Patton, 2015). A standardized study introduction invitation letter was sent via email and social media to numerous faith and statutory agency organizations, which were asked to share the communication with their members. To ensure wide U.K. distribution, the study information was sent to groups in Scotland, Ireland, and Wales as well as England (e.g., British Association of Social Workers, Association of Child Protection Professionals, the National Working Group Child Abuse Linked to Faith or Belief, Christian Forum for Safeguarding, Faiths Forum for London, Inter Faith Network for the UK, the national working group for abuse linked to accusations of witchcraft and spirit possession, the Cross Party Group on Adult Survivors of Childhood Sexual Abuse (Scottish Parliament), the Cross Party Group on Faith (Senedd, Wales), the Northern Ireland Council for Voluntary Action faith engagement group, the All-Party Parliamentary Groups Safeguarding in Faith Communities). The full list of contacts used is available from the research team. If participants wished to take part, they followed the link in the invitation communication to the online survey.

### Procedure

Upon entering the online survey, participants read the information sheet and gave informed consent. They were then asked to indicate whether they were a member of a statutory body (SS) or working within a religious organization (FBO). This question routed participants to the contextually specific questions for their group. Participants then completed Section 1, which contained demographic data. They then moved to the six open-ended questions and, finally, to a debrief section, which also included space for them to leave contact details if they wished to take part in a further follow-up study.

### Data analysis

Data were downloaded into an Excel file and analyzed using thematic analysis (Braun & Clarke, 2006), which included familiarization with the data, generation of codes, combining codes into themes, reviewing themes, determining significance of themes, and the reporting of findings. Downloaded answers were read and initial notes were made relative to the key aims of the study. They were then reread, and salient points were developed into codes. These codes were then interpreted, and any codes that shared commonalities were grouped together to create higher-order themes. For each code, quotations were extracted from the transcripts that highlighted the code and helped to clarify and demonstrate its meaning. Researcher triangulation occurred through the first author and third author discussing the coding and theme construction until they reached agreement.

## Results

### Participants

In total, 117 respondents engaged with the online questionnaire, of whom 89 provided qualitative responses to the questions asked. This study reports on the data from these 89 participants. See Table 1 for demographic information about participants. The mean age of participants was 52.42 (*SD* = 11.71). Seventy-eight participants worked within FBOs and 11 worked within SSs. Participants had worked in their current roles for an average of 6.01 years (*SD* = 4.82). Fifty-one (66.7%) of the 78 FBO participants noted that they had experience working for SSs. Most participants were female (72%), from England (88%), identified as Christian, and worked as faith-based safeguarding leads (88%). A small percentage identified as Jewish, Muslim, Buddhist, Humanist, or Unitarian.

**Table 1:** Participant demographics (*N* = 89)

Demographics		*n*	%
Organization	Faith-based organizations	78	87.6
	Statutory services	11	12.4
Sex	Female	64	71.9
	Male	25	28.1
Country	England	78	87.6
	Wales	3	3.4
	Scotland	2	2.2
	Northern Ireland	1	1.1
Religion*	Christian	66	84.6
	Atheist	3	3.8
	Jewish	2	2.6
	Muslim	2	2.6
	Buddhist	1	1.3
	Humanist	1	1.3
	Unitarian	1	1.3
	No religion	1	1.3
Role	Safeguarding lead	47	52.8
	Deputy safeguarding lead	12	13.5
	Senior leadership	7	7.9
	Safeguarding specialist	6	6.7
	Management	4	4.5
	Other	13	14.6
Paid/voluntary*	Paid	43	55.1
	Voluntary	35	44.9
Contact with FBOs/SSs	Yes	71	79.8
	No	18	20.2
Frequency of contact	0–5 times	26	29.2
	6–10 times	13	14.6
	10–15 times	4	4.5
	16 + times	28	31.5

*FBOs only. FBO = faith-based organization, SS = statutory service

### Themes

Four overarching themes were developed from the data: understanding and supporting spirituality, safeguarding awareness and challenges, communication and collaboration barriers, and suggestions for improved partnership (see Table 2).

**Table 2:** Themes and subthemes

Overarching Theme	Subthemes
Understanding and supporting spirituality	SSs are helpful
	SSs facilitate the spiritual needs of individuals
	SSs understanding the nature of spirituality
Safeguarding awareness and challenges	SSs’ lack of understanding of FBOs’ safeguarding roles
	SSs’ lack of understanding of the faith context
Communication and collaboration barriers	SSs’ lack of collaboration and information sharing
	SSs’ processes can be convoluted
	SSs can be patronizing and rude
Suggestions for improved partnership*	Joint training and increased open communication
	Improved training on specific safeguarding issues
	Encourage professional and respectful teamworking

*Participants who did not have contact with SSs/FBOs (*n* = 18) only gave suggestions for improved partnership. SS = statutory service, FBO = faith-based organization

#### Theme 1—Understanding and supporting spirituality

This overarching theme describes how stronger relationships are formed when SSs understand spirituality and the faith context. Forty-two participants (out of 71) reported *positive perceptions* when working with SSs/FBOs. One participant explained: “Once staff are assured of our role etc., we tend to find that statutory staff engage with us well” (safeguarding lead, Christian FBO in England). The first positive experience highlighted by FBOs is that *SSs are helpful.* One participant wrote: “Statutory agencies have been helpful by sharing information that will help us to assess risk in our own context and to make decisions about people who may cause harm or may be at risk themselves within our church communities” (safeguarding casework supervisor, Christian FBO in England). Other FBO participants (7 out of 71) explained that positivity from their working relationships with SSs improves when *SSs facilitate the spiritual needs of individuals.* One participant went on to explain that she was aware of probation officers who informed the church “that a certain offender is a Christian and wishes, upon release from prison, to attend X church... and then they work with the local church safeguarding officer to plan and set up a safeguarding contract” (safeguarding casework supervisor, Christian FBO in England). Finally, a few participants (6 out of 71) explained that positive experiences between SSs and FBOs stem from *SSs understanding the nature of spirituality*. One participant stated that when they are dealing with a designated contact within SSs, “The relationship and understanding of the role faith plays in people’s lives gets better and the capacity to work together and share information improves” (safeguarding lead, Christian FBO in England). In contrast, others gave examples of mixed experiences: “I can cite some fantastic examples of joint working—but also sometimes where I have felt ignored, let down, misunderstood, dismissed by statutory professionals” (safeguarding lead, FBO in England).

#### Theme 2—Safeguarding awareness and challenges

Within this theme, we describe how a lack of understanding of the faith-based context and of safeguarding roles can create problems. A key issue discussed by many participants (35 out of 71) was *SSs’ lack of understanding of FBOs’ safeguarding roles*. One participant discussed their experience: “Poor understanding of our role and function by some workers and adult social care. Frequently spoken to as if we do not have any safeguarding knowledge. Lack of understanding by statutory services about why we have referred” (deputy safeguarding lead, Christian FBO in England). A related issue discussed by FBO participants (26 out of 71) was *SSs’ lack of understanding of the faith context*. One participant explained: “My experience is based in statutory services having a lack of understanding about faith, having preconceived ideas about faith, [and] stereotypical views about faith” (safeguarding lead, Christian FBO in England). This was exacerbated when interactions were not based on evidence or fact. For example, “I was a little disappointed in communication that was based on gossip and negative assumptions which led to opportunities for disrupting abuse to be missed” (safeguarding specialist, Children’s Social Services in Scotland).

#### Theme 3—Communication and collaboration barriers

Another common issue that resulted in negative experiences for FBO participants (18 out of 71) was *SSs’ lack of collaboration and information sharing*, as exemplified in the following statement: “Often statutory services will not share information as they are concerned it would breach the person’s data protection rights so that we sometimes find it hard to risk assess” (safeguarding casework supervisor, Christian FBO in England). FBO participants (16 out of 71) also explained that *SSs’ processes can be convoluted*, making it difficult to progress casework. One participant explained: “Referring into Children’s Safeguarding is a very long process. You initially have to speak to the duty officer before being allowed to fill out the referral form. It is very frustrating if you just get the answerphone!” (safeguarding lead, Christian FBO in England). Finally, a small number of FBO participants (7 out of 71) discussed how *SSs can be patronizing and rude* to them. One participant remarked: “In the past I’ve found them to be rude, aggressive, and patronizing.... When they asked questions, I felt like I was giving the wrong answer.... It felt like they had their mind made up before going into the meeting” (safeguarding lead, Unitarian FBO in England).

#### Theme 4—Building better partnerships

Participants also offered insights into how the relationship between SSs and FBOs could be strengthened. The most common suggestion by participants (39 out of 89) was *joint training and increased open communication*. One participant suggested: “Perhaps there should be some joint training.... I think there needs to be an honest dialogue between the teams, to explain and understand each other’s roles and how we can support an investigation” (safeguarding advisor, Humanist church in England). As well as joint training, some participants (23 out of 89) also suggested *increased training on specific safeguarding issues*. One participant suggested “education for statutory colleagues regarding the role and function of safeguarding teams within faith contexts so they understand that we are professionals and can/should engage on the same terms as other professionals” (safeguarding lead, Christian FBO in England). Similar to joint training, a final suggestion (12 out of 89) was to *encourage professional and respectful teamwork*. One participant remarked: “Creating and maintaining conversations and dialogue is extremely important. This will ensure mutual interests are maintained and, as statutory organizations, we will have knowledge of what faith organizations expect from us” (senior mnager, Safeguarding Children Partnership in England).

## Discussion

The aim of this research was to explore the factors that facilitate or hinder effective collaboration between SSs and FBOs. Critically, despite a wide recruitment strategy, significantly more faith-based leads than those working within the statutory sector responded, and thus our discussion and results include more faith-based perspectives. Regardless, the findings from this study highlight the multifaceted relationship between safeguarding leads in FBOs and SSs. We begin our discussion by exploring the key themes from the data, considering the implications for policy and practice, and we then discuss how these align with the NPT and then propose strategies to enhance collaboration and effective working.

The majority of FBO participants reported positive experiences when working with SSs, identified through theme 1, particularly when SSs were helpful and understood the role of spirituality and the importance of facilitating the spiritual needs of individuals. It was also reported that positive experiences were more likely to occur when FBO safeguarding leads perceived the SSs to be helpful and worked to forge strong working relationships and when their contact understood spirituality. In line with the results of this study, early work by Morrison (2000) and Laming (2003) emphasized the importance of role clarity, coordination, and responsibilities in effective safeguarding collaboration. While only a few studies (Oakley & Kinmond, 2014; Oakley et al., 2018) have sought to establish the importance of such strong working relationships and the relevance of SSs’ understanding safeguarding matters related to spirituality (Oakley & Kinmond, 2014), several government reports and frameworks (HM Government, 2014, 2023) have already set out the need for greater collaboration between these groups, ultimately for the benefit of vulnerable individuals (Oakley & Kinmond,  2016).

However, almost half of the participants highlighted negative experiences (themes 2 and 3). The key issues included a lack of understanding of mutual roles, lack of faith context understanding, limited collaboration and information sharing, inefficient processes, and perceived rude and patronizing behavior. Research by Stevens (2013) and Gilligan (2012) indicates that such issues are common in interagency work and can significantly hinder effective collaboration and safeguarding. Many participants explained how SSs did not understand the role of spirituality and the relevance of the faith context, resulting in a poorer working relationship. This finding mirrors that of Sidebotham et al. (2016), who found that a common barrier to a healthy working relationship was the constant misunderstanding of roles. When SSs are not aware of safeguarding leads in FBOs, this can negatively affect the working relationship, which has a damaging effect on the individual who needs support.

Furthermore, as discussed in the findings, experiences of working with SSs who are unwilling to share information due to their misunderstanding of the faith setting role is another key barrier to good safeguarding practice. Similarly, participants noted that the complex processes and systems they need to follow can exacerbate issues surrounding working relationships, sometimes causing frustration. Moreover, both Morrison (2000) and Hood et al. (2016) allude to difficulties with bureaucracy and how this can often impact the individual’s ability to complete their work. In their paper which encourages the improvement of working relationships in these contexts, Hood et al. (2016) suggest that individuals should not only learn about the other organization’s roles but should also learn how to be open and receptive and willing to be respectful of others and recognize their roles. Doing so can also lead to positive outcomes for all parties.

Exploring the themes and subthemes through the lens of NLP allowed us to identify the facilitators and inhibitors to positive working relationships and implementation of best practice. The first stage of NPT coherence focuses on the sense-making work individuals do to understand a new practice, or in this case partnership working. From the results, coherence is reflected in subthemes such as “SSs’ understanding the nature of spirituality,” “SSs’ lack of understanding of the faith context,” and “SSs’ lack of understanding of FBOs’ safeguarding roles.” These highlight the importance of shared conceptual frameworks. Without mutual understanding, there is a risk to positive collaboration, a point noted by Hillis (2024), who states that coherence is foundational to building trust and legitimacy in cross-sector partnerships. From coherence we move to cognitive participation, which centers on the relational work required to engage individuals and groups in a new practice. Information from the subthemes “joint training and increased open communication” and “encourage professional and respectful teamworking” reflect efforts to foster engagement and commitment and what is or could be the development of the working infrastructure. When SSs and FBOs invest in joint learning and respectful dialogue, positive working collaborations can be established.

Collective action refers to the operational work of enacting a practice, and within the results there were examples of both positive and negative collective action. For example, the subthemes “SSs are helpful” and “SSs facilitate the spiritual needs of individuals” suggest that there is evidence of positive collective action. In contrast, the subthemes “SSs’ lack of collaboration and information sharing,” “SSs’ processes can be convoluted,” and “SSs can be patronizing and rude” identify examples of potential inhibition or breakdown of operational processes (Huddlestone et al., 2020).

The final stage, reflexive monitoring, involves the appraisal and adaptation of practices over time. Information from the subthemes “improved training on specific safeguarding issues” and “encourage professional and respectful teamworking” are relevant to this stage. Furthermore, to develop positive practices, both the FBOs and the SSs need to engage in reflective practice and shared feedback. This is crucial to ensure partnerships are sustained and those in development continue to evolve (C. R. May et al., 2025). Discussing the findings relative to NPT suggests that there are areas of good practice, although coherence and cognitive participation may be underdeveloped while collective action and reflexive monitoring reveal both operational strengths and areas for improvement.

The need to improve working relationships was a key area within the findings. Participants highlighted the following suggestions to improve the working relationship between FBOs and SSs: further training on safeguarding in faith-based contexts for SSs, increased open communication, professionalism, and respectful teamworking. The latter suggestions (professionalism and respectful teamworking) reflect an approach advocated by both Morrison (2000) and Hood et al. (2016), particularly in the context of safeguarding children. However, it also raises the practical issue of increased caseloads for SSs and the numerous faith contexts one individual may encounter. Indeed, many FBOs participants discussed being misunderstood by SSs; therefore, training on handling faith-based caseloads and the sensitivity surrounding them could be considered. One solution, from Pentaris’s (2019) perspective, is to increase religious literacy alongside providing training to reduce any avoidant or utilitarian approaches in practice, ensuring that SSs feel upskilled and able to ask questions related to faith in a way that respects all individuals’ views. Previous research by Oakley et al., (2016, 2018) has suggested that individuals from FBOs also require training in understanding their roles as safeguarding leads within FBOs and that any lack of understanding of roles (for example, of SSs) or process may hinder the working relationship. This could also possibly be done through a service user–lead approach which could involve actively seeking input and feedback from those who use these services to ensure that the training is practical, relevant, and sensitive to the needs of diverse faith communities. Together, these approaches echo those discussed by Morrison (2000), who stressed the utmost importance of good teamworking between FBOs and SSs to ensure the ultimate well-being of vulnerable individuals.

Joint training would allow the sharing of best practice and the development of collaborative working protocols This would allow for what Hood et al. (2016) terms the development of a “common language” shared between organizations. Furthermore, in line with the suggestion that increased open communication will improve partnership working, sharing in this way would foster positive relationships that would benefit all users, including those in need of support from safeguarding professionals. As Appleton (2012) states, collaboration is a critical feature of successful working within safeguarding, and respondents in their study believed that effective multiagency working was crucial to ensure positive outcomes for safeguarding. There is also a need to consider issues identified from the perspective of SSs. Although there was not enough data collected in this study regarding their views, researchers should focus on how statutory agencies collaborate with religious organizations to safeguard vulnerable individuals within those communities and protect them from potential abuse (Hood et al., 2016).

## Limitations and recommendations for future research

Although interesting findings emerged from the study, there are limitations. One key limitation to the study was the limited number of responses from SSs, which meant that we could not compare views as readily as anticipated. Thus, our data represent more of a one-directional perspective, highlighting the views of safeguarding leads in FBOs. The reasons for the limited response from SSs could be varied. It could simply be due to communication disparity. When FBOs need to discuss cases, they are more likely to speak to SSs, whereas SSs may not readily talk to FBOs. Another reason could be due to organizational contexts as FBOs have a direct stake in the importance of these working relationships with SSs, whereas SSs work with a much broader range of organizations in numerous contexts and therefore may not be as inclined to participate. Likewise, participating in our study may have had a greater impact on FBOs than SSs. Another possibility is that SSs were hesitant to participate, anticipating that discussions might involve caseloads they viewed as ethically or procedurally inappropriate. Although some participants in the study did identify as working for a SS, future research could directly target SSs alone for their views on these relationships. We also had more female than male respondents, which could be a result of the FBO safeguarding role being in many cases voluntary, and within the United Kingdom women make up the majority (68%) of the voluntary work force (National Council for Voluntary Organisations, 2024). Finally, we as the research team may have inadvertently biased data collection through the recruitment process. While we compiled a representative list, the fact that the email originated from an FBO may have influenced whether people chose to respond. Taking all these points into consideration, overall, FBOs may have been more likely to respond than SSs.

Critically, when looking at the data collected, while more participants reported positive experiences, they did not give as much detail about these, possibly due to the positivity-negativity asymmetry phenomenon (Baumeister et al., 2001) in which negative experiences have a larger impact and are thus remembered in more detail. Therefore, participants’ discussing more instances of negative experiences does not necessarily reflect real practice. While qualitative online surveys allow for wide reach and quick responses, they are reliant on the participant to provide detail from the prompts, without any room for further exploration of key issues. Thus, future research could look to capture richer data, for example, through using interviews with scripts co-created with representation from the groups under investigation (Vargas et al., 2022). Future follow-up research should ensure that there are prompts within any interviews to draw out details on positive experiences to ensure the data collected are representative of the whole picture and to capture good practices that aid positive working relationships. Finally, we do not know whether there is any regional or agency variation in these findings or whether the findings are also relative to specific faith settings. Importantly, future research should focus on context and culture and look toward country comparisons. All these factors are critical questions that, if answered, would also develop our understanding and contribute to best practice.

## Conclusion

These findings further underscore the complexity of the relationship between FBOs and SSs in safeguarding practice. While many positive aspects of good working practice between FBOs and SS were highlighted, significant challenges remain. The key issues highlighted by FBOs were faith illiteracy (meaning the role and function of FBOs not being properly understood), poor information sharing, inefficient processes, and not feeling properly understood (manifesting as experiencing rudeness and patronizing behavior). Enacting the participants’ suggestions of implementing targeted training, improving communication, and fostering respect and professionalism could significantly enhance the collaboration between FBOs and SSs, leading to more effective safeguarding practices and benefiting vulnerable individuals who need support. Using the NPT allowed us to frame the findings in relation to operational practice, and it also presents a mechanism through which those actively engaged in safeguarding can reflect on their practice.
